# Rights and Responsibilities of Tuberculosis Patients, and the Global Fund: A Qualitative Study

**DOI:** 10.1371/journal.pone.0151321

**Published:** 2016-03-21

**Authors:** Muhammad Atif, Sareema Javaid, Maryam Farooqui, Muhammad Rehan Sarwar

**Affiliations:** 1 Department of Pharmacy, The Islamia University of Bahawalpur, Bahawalpur, Punjab, Pakistan; 2 Faculty of Pharmacy, Universiti Teknologi MARA (Bertam campus), Persiaran Pendidikan Bertam Perdana, Kepala Batas, Penang, Malaysia; Public Health Agency of Barcelona, SPAIN

## Abstract

**Background:**

Implementation of the Charter to protect patients’ rights is an important criterion to achieve patient-centered approach and receive financial support from the Global Fund. Our study aims to explore the knowledge of tuberculosis (TB) patients about their rights and responsibilities at the Chest Disease Unit of the Bahawal Victoria Hospital, Bahawalpur, Pakistan.

**Methods:**

This was a qualitative study. The data from purposefully selected TB patients was collected by in-depth interviews. Eligibility criteria included confirmed diagnosis of TB and enrollment in the TB program. A pilot tested interview protocol was based upon the objectives of the study, and was used uniformly in each interview to maintain the consistency. The sample size was limited by applying the saturation criteria. All interviews were audiotaped and transcribed verbatim. Inductive thematic content analysis was applied to analyze the data and draw conclusions.

**Results:**

Out of the total 16 patients, four were female, and seven were illiterate. Eight patients were known cases of multi-drug resistant TB. Analysis of the data yielded seven themes; tuberculosis care services, moral support and stigmatization, dignity and privacy, complaints, fear of losing job, information sharing and compliance to the treatment plan, and contribution to eradicate TB. First five represented the rights section while latter two were related to the responsibilities section of the Charter.

**Conclusion:**

Discriminatory access to TB care services and the right to privacy were two major concerns identified in this study. However, the respondents recognized their responsibilities as a TB patient. To ensure uninterrupted investment from the Global Fund, there is a need to implement fair TB care policies which support human rights-based approach.

## Introduction

Tuberculosis (TB) is still a major setback for public health sector globally, infecting millions of people every year. Alongside human immunodeficiency virus (HIV), TB is the leading reason of death among populations suffering from infectious diseases [1]. As per World Health Organization (WHO) survey, 9.6 million people developed TB in 2014, out of which 1.5 million died [1]. Although, TB is a deadly issue for all the regions of the world but it majorly hits communities with low socioeconomic background. South-East Asia and Western Pacific regions alone bear 58% of global TB cases [1]. With a prevalence rate of 341 cases per 100,000 population, Pakistan stands fifth in the line of countries with high TB burden and shares 61% of the total TB cases in the Eastern Mediterranean Region [1,2]. In 2014, a total of 281,172 new and relapse TB cases aged ≥15 years were notified in the country, and the male to female ratio was 1.0 [1]. According to a recent report, Pakistan has met the Millennium Development Goal (MDG) target of 50% reduction in the mortality rate by 2015 compared with 1990. However, the targets of reduction in the incidence and prevalence rates are not met [1]. Pakistan is also in the queue of those countries having highest proportion (i.e., 3.7% of new TB cases and 18% of retreatment TB cases) of multidrug-resistant TB (MDR-TB) [1].

To control the high incidence and mortality rate around the globe, WHO in 2006 drafted the Stop TB Strategy which aimed to achieve the targets set by the MDGs [3,4]. The core objectives of the WHO Stop Strategy emphasize in achieving the patient-centered approach to TB treatment and easy access to high quality TB treatment on equality basis [4]. The patient-centered approach empowers the patients to contribute actively as an informed partner in decisions and activities related to TB diagnosis and management. The five principals of the patient-centered approach are; recognize patient rights, enable partnership, empower and activate patients and communities, engage all stakeholders, and monitor and document [5]. Empowerment of the patients and community is also in line with the International Standards for Tuberculosis Care (ISTC) which aims to protect the human rights of the patients to ensure the equality-based delivery of TB care services to everyone [4,6]. The Stop TB strategy also encloses the key concept that high quality TB care and treatment could be achieved by the joint efforts of the National TB Program (NTP) staff, TB patients (both cured and suffering) and the community [4].

Tuberculosis patients around the world have developed The Patient’s Charter for Tuberculosis Care (the Charter) [7]. The Charter supports the ISTC to implement the patient-centered approach [6,7]. The Charter provides a firm base to protect the rights of poor and vulnerable TB patients to relieve them from socioeconomic burdens associated with this disease. It also holds TB patients as much responsible as the healthcare providers for their treatment [7]. The published evidences have revealed that human rights-based approach increased the efficacy, effectiveness and sustainability of malaria, TB and HIV programming [8,9].

The Global Fund, a Switzerland based financial institution, provides support to countries with poor socioeconomic profiles to fight against acquired immunodeficiency deficiency syndrome (AIDS), TB and Malaria [10]. Until now, the Global Fund has disbursed US$ 262,499,652 in Pakistan to fight against TB, malaria and HIV. Out of this, US$ 181,491,626 (69.1%) has been disbursed on TB program [11]. For the applicant countries, the Global Fund’s new funding model (2012–2016, strategic objective 4) has made it explicit to provide evidence for the treatment with protected human rights to ensure the sound programming [12]. The Global Fund has drafted a Tuberculosis and Human Rights Information Note which describes the importance of human rights in TB treatment [12]. In accordance with this note, the funding preference will only be given to the programs which will develop strategies to minimize human rights-related barriers to TB care. Furthermore, no support will be extended to the programs which violate and defy human rights in TB care and treatment [12]. Despite of such importance being given to human rights-based approach, limited literature is available on this aspect of TB care and treatment. For example, an Iranian study demonstrated that the TB patients were concerned about the behaviour of healthcare workers, privacy of their information and the financial consequences of TB [13]. Similarly, in a Nepalese study, the patients were not satisfied with the level of care given by the healthcare providers, and they experienced discrimination during their interaction with the wider community [14]. However, these studies [13,14] were unlike to the topic under discussion in terms of their objectives and outcomes.

In response, we designed a qualitative study based upon patients’ basic rights and responsibilities mentioned in the Charter. The findings will help the NTP staff to evaluate whether TB care providing centers are integrating human rights considerations in their routine practices to ensure that each TB case is treated according to the ISTC guidelines, and there is uninterrupted and increased investment from the Global Fund. The aim of this study was to explore the knowledge of TB patients about their rights and responsibilities in Pakistan.

## Methods

### Study setting

The study was conducted at the Chest Disease Unit (CDU) of the Bahawal Victoria Hospital (BVH) which is situated in the Bahawalpur district of the Punjab province, Pakistan. This hospital is above 1500-bedded, fully equipped, tertiary-care hospital with all medical and surgical specialties, serving a large number of patients in the Southern Punjab. It has well established and well equipped chest and TB ward catering both indoor and outdoor patients.

At the CDU, 8–10 physicians, 5–6 chest specialists and two pharmacists are providing routine care to the patients suffering from various chest-related diseases. About 35–40 TB patients (all forms) visit the CDU daily to take their TB therapy. Currently, about 80 active MDR-TB cases are managed in a separate section of the CDU. The TB section of the CDU is funded by the Global Fund and the NTP.

### Study design

The data for this qualitative study was collected by in-depth interviews [15,16]. The interviews were conducted from each patient in the premises of the BVH at a pace comfortable for the patients. A pilot tested interview protocol was based upon the objectives of the study, and was used uniformly in each interview to maintain the consistency [15].

### Sampling and data collection

The study was conducted between February and March 2015. The participants were aged 18 years or above having confirmed diagnosis of TB and had enrollment in the TB program. Purposeful sampling technique was employed to look for the information rich cases [17]. Sampling of the patients was done irrespective of the type of TB while final selection depended upon consent of the patients. The sample size was limited by applying saturation criteria [18].

Two members of the research team conducted the interviews in the premises of the BVH [19]. Two other members of the research team were involved in tape recording and taking the field notes. All interviews were conducted in the national language of Pakistan (i.e. Urdu). The patients we who were unable to understand Urdu (national language of Pakistan) were excluded from the study. Before starting the interviews, a friendly relationship was established with the consented participants. They were explained about the purpose of the study, and were ensured that the interview will not affect their treatment plan. After the completion of each interview, the respondents were offered either to listen the recording of their interview or to read the transcript on the next visit.

### Data analysis

Inductive thematic content analysis was applied to analyze the data and draw conclusions from the emerging themes. All interviews were translated from Urdu to English word by word. After translation, *decontextualization* method was applied to the translated data. The audio recordings were listened and the noted observations were read by all researchers again and again for each individual case. After a detailed discussion, the meaningful data was separated from each individual case and relevant codes were assigned to them [20]. *Recontextualization* was then applied to the coded data in which the coded data was analyzed and examined to reduce and organize the same information into broader subcategories [20]. These subcategories were further analyzed and summarized into categories according to the aims and objectives of the study. The drawn themes and conclusions were studied again and again by each researcher to ensure that it reflects the aims of our study. Discrepancies were discussed among the research team members to reach a final consensus. At the end, an individual researcher who did not participate in the data collection process examined the material to ensure validity.

### Ethical considerations

The study was based on the Code of Ethics of the Declaration of Helsinki. The ethical approval was taken from the Pharmacy Research Ethics Committee (PREC) at the Islamia University Bahawalpur (Reference: 107-2014/PREC, dated December 10, 2014). Before starting the interviews, the data collectors explained the purpose of the study to the target participants. We obtained verbal consent from the agreed participants. Written consent was not possible for most of the respondents either because they were illiterate or they had problems in reading and/or signing the consent document. The PREC committee approved the verbal consent procedure. The patients were assigned alphabets (i.e., Patient A, Patient B, etc.) rather the names to keep their confidentiality. All patients were given the right to leave the interview at any time during the interview process. The audio recorded interviews were deleted after the final step of the study and were not used for any other study. In this article, we replaced the phrases ‘suspected TB patients’ with ‘patients with possible TB’ and National Tuberculosis Control Program’ with ‘National Tuberculosis Program’ in an effort to avoid the use of *inappropriate* language in TB services [21].

## Results

A total of 16 interviews were conducted. Eight patients were known cases of MDR-TB. The interview duration ranged from 15–29 minutes with a mean duration of 25 minutes. The characteristics of the individual respondents are given in Table 1. The majority of the respondents did not show complete and clear understanding of their rights. Perceptions of a few patients about their rights were totally different from those mentioned in the Charter.

**Table 1:** Characteristics of the patients.

Patient	Age	Gender	Type of the patient	Employment status	Educational status	Interview duration (minutes*)
A	60	Male	MDR-TB	Employed	Illiterate	25
B	32	Male	MDR-TB	Employed	Primary	23
C	50	Female	MDR-TB	Housewife	Illiterate	27
D	55	Male	MDR-TB	Business	Illiterate	28
E	29	Male	DS-TB	Business	Primary	29
F	65	Male	DS-TB	Business	Secondary	29
G	19	Male	MDR-TB	Student	Diploma	25
H	30	Female	DS-TB	Housewife	Illiterate	28
I	25	Female	MDR-TB	Housewife	Tertiary	20
J	27	Male	DS-TB	Employed	Secondary	22
K	24	Male	DS-TB	Business	Primary	21
L	45	Male	DS-TB	Business	Secondary	26
M	25	Male	MDR-TB	Business	Illiterate	27
N	40	Male	DS-TB	Business	Illiterate	15
O	42	Male	MDR-TB	Farmer	Illiterate	19
P	24	Female	DS-TB	Housewife	Primary	28

*Rounded, MDR-TB = Multidrug-resistant tuberculosis, DS-TB = Drug sensitive tuberculosis.

Inductive thematic analysis of the data yielded 22 categories and seven themes; five themes represented the rights section while two were related to the responsibilities section of the Charter (Table 2).

**Table 2:** Themes and categories of patients’ rights and responsibilities.

**Theme**	**Categories**
**Rights**	
**Tuberculosis care services**	Behaviour of the healthcare professionals was unsatisfactory.
	Access to all medicines was not free.
	The patient required references to seek the maximum attention of the physicians.
	The patients were willing to seek a second medical opinion.
	There was a lack of concordance.
	The physicians provided insufficient drug-related information to the patients.
	Outpatients had access to their medical reports and charts.
**Moral support and stigmatization**	The patients received moral support from the family members.
	The patients observed negative changes in the behaviour of peers.
**Dignity and privacy**	The healthcare professionals respected the dignity of the patients.
	The patients were unaware whether confidentiality about their disease is maintained or not.
	The physicians should ask the patients before sharing their disease-related information.
**Complaints**	The patients did not know to whom they should complain.
	In case of a complaint, the patients may face negative behaviour of the physicians.
	The patients intended to complain in case they faced any care-related problem.
**Fear of losing job**	Employed patients were worried about rejoining their jobs.
**Responsibilities**	
**Information sharing and compliance to the treatment plan**	The patients were aware that they should provide all disease-related information to the physicians.
	The patients were aware that they should follow the treatment plan.
	Poor financial status was a barrier to follow nutrition-related advice.
	The patients were unable to resist their temptations.
**Contribution to eradicate tuberculosis**	The patients did not have sufficient information about tuberculosis care organizations.
	The patients were interested to participate in the tuberculosis eradication programs.

### Rights

#### Theme 1: Tuberculosis care services

Expectations of the patients from the healthcare professionals about the level of care were generally not up to the standards as mentioned in the ISTC. The behaviour of the healthcare professionals towards the patients was not equal as not all patients were satisfied with the attention they were receiving from the healthcare staff. Only four patients claimed that they were getting free medicines from the hospital. Some patients told that they were not getting free treatment as per the guidelines of the NTP of Pakistan while some patients told that they were getting fewer medicines free of cost from the hospital and have to buy other medicines on their own. They were told by the healthcare providers that there was a shortage of medicines in the hospital.

*“We know that treatment of TB is free of cost*, *but we buy our own medicines*. *Doctors say that there is a shortage of medicines in the hospital since long”*. (Respondent I)

Some patients complained that they were not treated well by the physicians and the nurses and the physicians did not discuss with them in detail about their disease due to a large number of patients. Two patients were even of the view that the only way to get maximum attention by the physicians was to consult them with some reference.

*“No one gives you attention without a reference*. *We have to wait in long queues*, *but those who come with a reference manage to meet the doctors first*. *I also came here by the reference that is why my turn came early”*. (Respondent G)

Some patients reported that, in case of no clinical improvement, it was better to take a second medical opinion. However, they were unable to opt for this either because their current physicians did not like it or they were financially poor.

*“One should take advice from two to three doctors*, *but I am poor and cannot afford the doctor’s heavy fee that is why I cannot go”*. (Respondent L)

Some patients even believed that whatever therapy plan they were receiving was the only correct therapy existed, no matter they felt comfortable with it or not. Most of the respondents stated that injectable were painful but they have to follow the treatment plan because either there was no alternative therapy plan or due to lack of concordance between the physicians and the patients.

*“I do not like taking medicine via injection*, *but doctor do not ask how you want to take your medicine*. *They injected medicines four times today in the morning which caused a lot of pain*, *but maybe there is no other way available and it is better for us to follow whatever the doctor is saying”*. (Respondent I)

The majority of the patients reported that the physicians did not give them much information about the medicines for example their side effects, normal actions, etc. However, the physicians advised the patients to follow their treatment plan.

*“Doctor told me that it has treatment course of 9 months*. *Doctor asked me not to take fatty meal and also to take cold water*, *he told me all the safety measures*. *Doctor also told me the time to take medicines; he told me to take medicine 15 minutes before breakfast”*. (Respondent D)

*“Doctor do not tell about the side effects ever*. *I do not know what medicines do after going inside our body”*. (Respondent C)

Most of the patients told that the physicians informed them about their disease and reports and they were allowed to access their medical reports and charts. But the inpatients were not allowed to take a copy of their medical charts with them.

*“Chart on which doctor writes medicines is with me*, *but sometimes do not know why nurse take it from me*. *Once I asked for my medical chart because I had to consult some other doctor*, *but she refused to give it to me”*. (Respondent M)

*“My reports are always with me; whenever I come here I take them with me”*. *(Respondent L)*

#### Theme 2: Moral support and stigmatization

The patients suffering from TB were supported morally by their family members. The majority of the patients reported that they did not find any negative changes in the behaviour of their family members when they knew about their disease and did not let patients feel any sort of discrimination and hatred. The family members encouraged the patients to take their medicines on time and showed their kindness and sympathy.

*“Behaviour of family members is really good with me*. *I have four kids*, *everyone supports me and my wife takes care of me a lot and gives me medicines with responsibility*. *Behaviour of other family members is also very good with me*. *I did not have money; they are bearing my treatment expenses”*. (Respondent M)

Some patients noted negative changes in the behaviour of peers. They thought that peers and relatives started maintaining a distance when they came to know about their disease.

*“I feel bad when people stay away from me*. *When people get to know that I am a TB patient then they do not say anything from the mouth*, *but in their hearts they do say that I should stay away from them*. *I*, *myself maintain a distance”*. (Respondent L)

#### Theme 3: Dignity and privacy

The dignity of the patients was respected by the healthcare professionals. The majority of the patients reported that they did not feel disrespected in the hospital and the healthcare professionals did not ask humiliating questions.

*“Here everyone communicates less*, *but whatever they say*, *they say it in a good way*. *They never ask any question which hurt us”*. (Respondent N)

The majority of the patients reported that they did not know whether the staff in the hospital shared their medical condition and other information with others or maintained the confidentiality. They said that it was physicians’ right whether to keep their information confidential.

*“It is doctor’s will whether he wants to share our information with any other person or not*. *I never find it necessary to ask the doctor not to tell anyone about my disease”*. (Respondent J)

However, some patients reported that the physicians should ask and take permission from the patients before sharing their information with any other person.

*“All of our information should be given to others with our consent; it is possible that I do not want to tell people about my illness or to any other doctor who is not good”*. (Respondent P)

#### Theme 4: Complaints

Most of the patients told that they did not know to whom they could complain if they were facing any problem during their treatment. They reported that they would not like to complain as they were having some sort of fear from the healthcare professionals. One patient reported that when he made a complaint, the authorities took action against him rather than listening to him so he stopped complaining.

*I do not know where to launch a complaint*. (Respondent A)

*“When I was taking treatment from the village*, *doctors’ behaviour was not good to me there*. *I made a complaint against them*, *but in return they filed a complaint against me to higher authorities so I get quiet”*. (Respondent M)

Three patients were of the view the patients should complain in case if they faced any problem during their therapy.

*“If I will be having any complaint regarding my treatment then I will surely tell*. *I will tell it to medical superintendent*, *doctor with high authorities is also my friend”*. *(Respondent F)*

#### Theme 5: Fear of losing job

The self-employed patients reported that they would continue their business after completing their treatment. However, the employed patients were confused whether they would be able to rejoin their respective job or they would be fired from the job due to a history of contagious disease.

*“Do not know that I can go back to my work or not*, *I did not even tell anyone there about my illness*. *May be I go back to work after I get better and they will not fire me otherwise will find some other work”*. (Respondent A)

*I will get back to my work*. (Respondent K)

*I will continue my work*. *We have our small family business*. (Respondent N)

We have summarized the findings of the rights section in the context of the Global Fund’s five minimum human rights standards (Table 3). Discriminatory access to TB care services and the right to privacy were two major concerns identified in this study. The study’s findings also identified lack of concordance between the physicians and patients.

**Table 3:** The Global Fund minimum human rights standards.

Human rights standards	Findings
**Non-discriminatory access to services for all, including people in detention**	The patients reported discriminative behaviour of the healthcare professionals. The patients believed that they can get maximum attention of the healthcare professionals by consulting them with a reference. The healthcare professionals did not provide sufficient drug-related information to all TB patients. The healthcare professionals did not like taking second medical opinion.
**Employing only scientifically sound and approved medicines or medical practices**	Only standard medicines were available at the clinic. However, in this paper, we did not evaluate guidelines adherence of the healthcare professionals.
**Not employing methods that constitute torture or that are cruel, inhuman or degrading**	None of the patients reported any cruel or inhuman treatment practice. However, there was a lack of concordance between the physicians and patients. Some patients reported pain associated with injection.
**Respecting and protecting informed consent, confidentiality and the right to privacy concerning medical testing, treatment or health services rendered**	The healthcare professionals respected the dignity of the patients. The patients were unaware whether the confidentiality of their medical condition was respected. The patients emphasized the need to take their consent before sharing their medical condition.
**Avoiding medical detention and involuntary isolation, to be used only as a last resort**	The patients did not report any problem associated with medical detection. Multidrug-resistant tuberculosis cases were isolated during initial days of the treatment.

### Responsibilities

#### Theme 6: Information sharing and compliance to the treatment plan

Most of the participants were of the view that the patients should provide all relevant information to the physicians. Similarly, the patients reported that the physicians should ask everything from the patients for the proper diagnosis of the disease, and prescribing of the medicines.

*“We should tell everything correctly to the doctor because he is a doctor*. *If we tell doctor only then he will consider everything and prescribe a medicine*. *If the doctor does not know about the disease*, *then how will he prescribe a medicine”*. (Respondent B)

Most of the participants reported that they themselves were responsible for their treatment and it was their duty to follow the instructions of the physicians. They reported that it was good to ask the physicians about their queries and discuss about their problems because to follow the treatment plan effectively it was important to understand it completely. They said that they were adherent to their treatment strategy.

*“I am responsible for my own treatment*. *If I have to get better then I have to take responsibility of the treatment*. *If I do the treatment completely*, *then I will be able to do anything in my life ahead”*. (Respondent D)

However, some patients reported that due to the poor financial status, they were unable to follow the physicians’ instructions about maintaining a good diet.

*“I took my medicines regularly*, *but I did not have enough money to maintain a good diet*. *I think due to poor diet*, *medicines did something wrong with me”*. (Respondent P)

Some patients also reported that they were aware that they should follow all instructions, but they were unable to resist their temptations.

*“I know if I quit smoking today I will get better today*, *but how can I quit? By the way I kept on reducing the number of cigarettes per day*. *Now I smoke 12 cigarettes per day*, *before I use to smoke 40 cigarettes per day”*. (Respondent F)

#### Theme 7: Contribution to eradicate tuberculosis

Most of the patients were aware that they should maintain a distance from the people to decrease the chances of disease transmission. They thought that in this way, spread of disease could be controlled.

*“I*, *myself maintain distance from my family*, *I sleep separately and sit separately*. *I have three kids*, *I maintain my distance from them as well*, *and even I do not show my love for them so that they do not catch this disease*. *I do not even touch them and do not let them use my utensils”*. (Respondent A)

The majority of the participants stated that they wanted to serve the community suffering from TB and were willing to collaborate with the healthcare providing authorities and other TB organizations. However, they did not have sufficient information about TB care organizations.

*“I want to do a lot of work*, *but I do not think so that we have such opportunities in our country or city*. *Till now I have never heard of any such organization where we can go and work for the betterment of other TB patients”*. (Respondent F)

Most of the patients reported that they would help the patients with possible TB in the community by convincing them to seek the medical advice as early as possible. The patients told that they would guide other TB patients about their treatment plan and precautionary measures.

*“Whatever doctor told me I will tell it to other TB patients that how to follow the treatment and which precautions to take so that they can also get their treatment done in a better way and this disease can end from our country”*. (Respondent E)

## Discussion

Our qualitative study aimed to evaluate the awareness of TB patients about their rights and responsibilities. We extracted 22 categories and seven themes. Five themes represented the rights domain while two themes characterized the responsibilities domain. With regard to the rights domain, there were issues related to TB care services, behaviour of the community towards TB patients, complaint procedures and job security. In terms of responsibilities, the patients were aware that they should provide disease-related information to the healthcare providers and have to follow their treatment plan. Moreover, they were willing to contribute in the efforts to eradicate TB. Nevertheless, some patients were unable to resist their temptations such as cigarette smoking. Some patients were unable to follow the dietary instructions because of their poor financial status.

### Rights

Free and equitable access to TB services is important to eliminate barriers to avail TB care services in an effective way [7,22]. Our findings clearly reflected that all patients were not satisfied with the care being provided to them at the hospital. Similarly, the patients were unaware of the alternative treatment options available to them and their right to choose or decline the proposed treatment plan. The patients were well aware about the availability of free of cost medicines but still they had to buy the palliative therapy from the market which was a financial burden for them. Similarly, the patients thought of consulting the physicians privately but again their poor financial status was acting as a barrier. A South African study also reported that the TB patients were financially compromised but they were well aware of the alternative treatment options available and they even thought of taking the herbal treatment rather than the allopathic treatment [23]. In contrast to our findings, a study conducted in South East Brazil reported that TB did not affect patients’ financial status as the treatment was totally free of cost [24].

The Charter and the Global Fund actively support patients’ right to proper and complete information about their disease and treatment [7,25]. It leads to enhanced compliance of the patients to the treatment strategy. The findings of our study indicated that the information provided to the patients by the healthcare staff needs improvement. The patients were not provided complete information about the prescribed medicines which is similar to the findings of a Nepalese study [14]. Sometimes, the patients consider the common side effects of TB medicines as harmful and dangerous, and they stop taking the medicines. This may lead to poor compliance, and emergence of MDR-TB is inevitable. The Working Document on TB and Human Rights recognizes right to information as an important part in increasing access to quality TB diagnosis, treatment, care and support [22]. Complete knowledge about TB medicines could eradicate misconceptions from patient’s mind regarding safety and efficacy issues and the patient feels more comfortable and adhered to the treatment plan. Therefore, there is a need to establish a therapeutic relationship between the healthcare workers and patients in which patients are encouraged to ask questions about their treatment plan.

Moral and emotional support from friends and family makes patients emotionally strong and helps them to complete their treatment plan without hurdles with a belief that they are not alone. Our study indicated strong family support being given to the patients although some patients reported that they noticed negative changes in the behaviour of their relatives and peers towards them. Various literature reinforced this finding that family and friends supported the patients mentally, emotionally and sometimes even financially [24,26]. However, a study from Zambia stated that sometimes family members and colleagues of TB patients started avoiding them because they were worried about catching the disease [27]. Negative changes in the behaviour of family members and peers lead towards stigmatization and social isolation of the patients [24,27]. Sometimes, the patients intentionally do not visit the physicians regularly as they do not want to be seen by others as a patient with possible TB. Similarly, fear of hatred by others mostly results in patients not adhering to the treatment plan openly and confidently which can lead to poor patient compliance. All these factors may lead to a patient becoming MDR-TB case which in turn is a burden on national economy in terms of cost involved in long-term treatment plan.

Due to social stigma, TB patients are always conscious about their privacy and eager to utilize healthcare services only when they are sure that their confidentiality will not be compromised [12]. The patients sometimes hesitate in describing their disease to the physicians because they do not know whether their disease will be kept confidential or not which leads to improper diagnosis and improper prescribing. According to our findings, the patients were not aware about their right to privacy and they were not provided easy access to their medical charts. They thought it as physician’s right whether to breach their confidentiality or not. According to a study conducted in South Africa, the patients even avoided going to TB clinics because they thought that their privacy was compromised [23].

Feedback from the patients is important for the proper implementation of the policies made by the NTP. According to our findings, the patients did not know a proper channel for filing their complaints. The Global Fund requires that the applicant countries should monitor the proper implementation of the policies regarding human rights [12].

Fear of no financial protection tortures the patient as TB is mainly a disease of poor community that lacks quality health life. This mental torture can encircle TB patients in more grief and they lose hope of getting normal and happy life back again. In this study, the employed patients were confused that whether they would be able to rejoin their jobs. Right to social security and financial protection aims to relieve patients from the financial stress which might be the consequence of unemployment either due to physical weakness or due to forced expulsion form the job due to contagious nature of the disease [22].

### Responsibilities

According to the Global Fund, TB can never be understood by the patients if they are not actively involved in planning and implementation of the policies [12]. A trustworthy and truthful relationship between TB patients and the healthcare staff is an important tool to involve the patients actively in their treatment plan. Our findings supported other literature that little confidence in healthcare staff always discourages patients to ask questions about the aspects of the treatment they were unclear about [14,28]. However, the patients were willing to share as much information regarding their disease with the healthcare staff as possible; they wanted long and detailed discussion sessions with the physicians to fulfill their responsibilities as patients. But lack of time and failure to develop unimpeachable relationship between the patients and physicians shook patients’ confidence which could be a strong barrier to identify TB patients as stakeholders in managing their disease as mentioned in the Charter [7].

A strong community build-up to fight against TB is only possible when the patients respect the rights of other patients too. Proper empowerment of TB patients involves guiding them about their rights and their due responsibilities in managing their disease. Our respondents showed zealous and fervent attitude towards community participation in helping other TB patients which is in accordance with the study conducted in Belgrade [29]. TB patients seeking support at least from other TB patients is none less than a streak of hope especially if they are the victims of social stigmatization. Exchange of experiences within TB community improve patients’ understanding of the disease and uplift their confidence when they hear success stories of other TB patients who are getting better and living their normal lives without any discrimination.

The Global Fund, the Stop TB Strategy and the WHO guidelines all revolve around the human rights-based approach to the treatment, prevention and cure of TB. Counseling of the patients about human rights-based approach to TB, and designing and initiating programs which aim to reduce human rights associated barriers [30] without discrimination is important so that no TB case could leave undiagnosed and the diagnosed cases are treated according to the ISTC. The supportive behaviour of family members is an important tool to motivate TB patients to complete the treatment in an effective way, and it will save the patients from social isolation. Special emphasis should be given by the healthcare staff to maintain the confidentiality of the patients. Each and every decision about the treatment should be made after obtaining consent of the patient, which is a key point of enhancing confidence of the patient over the healthcare staff. This enhanced confidence of the patient will increase the comfort level between the patient and the prescriber and the patient will feel more obliged to follow the treatment plan. The NTP should prepare an investigation team to ensure that the patients’ complaints are dealt in an effective manner. Translating the Charter in native language and guiding the patients about its content could also engage them as the stakeholders in their effective treatment. Our findings also demand the NTP of Pakistan to be keen and strict in implementing the policies made by WHO and the ISTC to achieve the MDGs. The funding policies of the Global Fund strongly encourage countries to design and implement strategies with protected human rights and it demands from the countries putting the funding request, a strong and authentic evidence of ensuring human rights-based approach. Pakistan, being a low socioeconomic country needs the Global Fund support to achieve the MDGs and for fulfilling the compulsory requirements, protected human rights approach is necessary.

In this study, we did not interview the healthcare professionals and the caregivers of TB patients about their views on the rights and responsibilities of TB patients. Their perspective is important and could answer most of the questions raised in this study. However, this investigation was beyond the scope of this paper and could be considered in future studies on this topic.

## Conclusion

The patients lack a complete understanding of their rights, however, they recognized their responsibilities. Discriminatory access to TB care services and the right to privacy were two major concerns identified in this study. Our findings also identified lack of concordance between the physicians and patients. Lack of understanding of patients’ rights is a barrier to effective TB management pivoted on human rights-based approach. The policies which support the protection of human rights tie a strong knot with the fund releasing policies of the Global Fund.

## Supporting Information

S1 File Interview protocol. (DOCX)
