# Supplementary material for: Rights and Responsibilities of Tuberculosis Patients, and the Global Fund: A Qualitative Study
Source: PLoS One. 2016 Mar 21;11(3):e0151321. doi: 10.1371/journal.pone.0151321 (PMC4801178; doi:10.1371/journal.pone.0151321)
Supplement: S1 File — (DOCX) [file pone.0151321.s001.docx]

**INTERVIEW PROTOCOL**

| **Discussion Topics** | **Examples of Specific probes** |
| --- | --- |
| **RIGHTS** | |
| **How do you feel about your disease?** | - How did you react when you first find out about your disease? Were you reluctant in telling others about your disease when you first find out? If yes, why? |
| **How do you perceive the attitude or behavior of health care providers towards you?** | - How much you are satisfied with the level of care given to you by health care providers? Did you face any difficulties? - Does every patient here get equal treatment protocols? If yes then how? |
| **How did your family members and peers behaved when they got to know about your disease?** | - What kind of changes did you notice in their behavior? Were they started avoiding you? If yes, how? |
| **How much you know about your treatment plan?** | - Are you comfortable with the treatment plan you are receiving? Do you share your experiences with other patients or vice versa? How does the staff here in hospital behave when you try to interact with other TB patients? |
| **What are your views about consulting more than one doctor at a time?** | - Have you ever talked about consulting to some other doctor for second opinion with your current doctor? If yes, what? How did your doctor behave or felt about taking second medical opinion? |
| **Do you feel insecure during your treatment?** | - How do you feel about your privacy and confidentiality during your visit to this healthcare facility? - What do you think about sharing your information with others by staff here without your consent? - Are you allowed to keep the copy of your medical charts and reports? - To whom you will consult if you feel any unfair practices going on within this healthcare facility? |
| **How did the people or authorities at your work place behaved when they got to know about your disease?** | - Were you given medical leaves by authorities at your workplace or had they taken your resignation? If yes, then on what grounds they got your resignation? |
| **RESPONSIBILITIES** | |
| **Are you comfortable in sharing your personal information with healthcare providers?** | - What are your views that it is right to ask personal information from the patients? |
| **How much you feel responsible in following your treatment plan?** | - Do you ask your healthcare providers if you are not clear about your treatment plan? - What are your approaches if you feel any difficulty in following your treatment plan? |
| **What suggestions will you give to any person if he/she is diagnosed with TB?** | - Will you refer that person to any doctor or will you tell healthcare providers about that person? - Are there any other precautionary measures you want to suggest? |
| **What will you do after completing your treatment?** | - Do you think you can be beneficial in any manner toward other TB patients? If yes, how? - Do you have any knowledge about this disease before or have you ever heard about “Stop TB campaigns” in your area or anywhere on social media e.g. television, newspaper, internet etc? - What will you say except this? |
